# Supplementary material for: Association of sociodemographic factors, lifestyle behaviors, anthropometric measures, and cardiometabolic health markers with blood pressure in adolescents: a cross-sectional analysis
Source: PeerJ. 2025 Oct 13;13:e20151. doi: 10.7717/peerj.20151 (PMC12530200; doi:10.7717/peerj.20151)
Supplement: Supplemental Information 2 — All codes used in raw data to identify all variables. [file peerj-13-20151-s002.pdf]

English-language Codebook		
Portuguese	English	
NOME	NAME	
IDADE	AGE	
IDADE_CAT	AGE_CATEGORISED	
SEXO	SEX	
TURNO	EARLY_SHIFT	
ZONA	LIVING_AREA	
ESC.RESP.	PARENTS_SCHOOLING	
ESC.RESP_DIC	PARENTS_SCHOOLING_DICHOTOMIZED	
UT.DOM1	DOMESTIC_UTENSILS_1_TELEVISION	
UT.DOM2	DOMESTIC_UTENSILS_2_RADIO	
UT.DOM3	DOMESTIC_UTENSILS_3_BATHROOM	
UT.DOM4	DOMESTIC_UTENSILS_4_AUTOMOBILE	
UT.DOM5	DOMESTIC_UTENSILS_5_DOMESTIC_WORKER	
UT.DOM6	DOMESTIC_UTENSILS_6_WASHING_MACHINE	
UT.DOM7	DOMESTIC_UTENSILS_7_DVD	
UT.DOM8	DOMESTIC_UTENSILS_8_REFRIGERATOR	
UT.DOM9	DOMESTIC_UTENSILS_9_FREEZER	
UT.CLASS	DOMESTIC_UTENSILS	
UT_CAT	DOMESTIC_UTENSILS_CATEGORISED	
UT_CAT_DIC	DOMESTIC_UTENSILS_DICHOTOMIZED	
PARQ1	PARQ_1	Physical Activity Readiness Questionnarie_Question_1
PARQ2	PARQ_2	Physical Activity Readiness Questionnarie_Question_2
PARQ3	PARQ_3	Physical Activity Readiness Questionnarie_Question_3
PARQ4	PARQ_4	Physical Activity Readiness Questionnarie_Question_4
PARQ5	PARQ_5	Physical Activity Readiness Questionnarie_Question_5
PARQ6	PARQ_6	Physical Activity Readiness Questionnarie_Question_6
PARQ7	PARQ_7	Physical Activity Readiness Questionnarie_Question_7
SAÚDE	SUBJECTIVE_PERCEPTION_OF_HEALTH	

SAÚDE_DIC	SUBJECTIVE_PERCEPTION_OF_HEALTH_DICHOTOMIZED	
AF1	AEROBIC_ACTIVTY	
AF1_DIC_2	AEROBIC_ACTIVTY_DICHOTOMIZED	
AF2	MUSCLE_STRENGTHENING_ACTIVITY	
AF2_DIC_2	MUSCLE_STRENGTHENING_ACTIVITY_DICHOTOMIZED	
TELA1	SCREEN_TIME_1	
TELA1_DIC	SCREEN_TIME_1_DICHOTOMIZED	
TELA1_DIC_2	SCREEN_TIME_1_DICHOTOMIZED_2	
TELA2	SCREEN_TIME_2	
TELA2_DIC	SCREEN_TIME_2_DICHOTOMIZED	
TELA2_DIC_2	SCREEN_TIME_2_DICHOTOMIZED_2	
TELA3	SCREEN_TIME_3	
TELA3_DIC	SCREEN_TIME_3_DICHOTOMIZED	
TELA3_DIC_2	SCREEN_TIME_3_DICHOTOMIZED_2	
TELA4	SCREEN_TIME_4	
TELA4_DIC	SCREEN_TIME_4_DICHOTOMIZED	
TELA4_DIC_2	SCREEN_TIME_4_DICHOTOMIZED_2	
SONO1	SLEEP_DURATION_1	
SONO2	SLEEP_DURATION_2	
SONOCLASS	SLEEP_DURATION_CALCULATED	
SONO_CAT	SLEEP_DURATION_CATEGORISED	
TAB_CAT	TOBACCO_CATEGORISED	
BEB	ALCOHOLIC_BEVERAGE	
BEB_DIC	ALCOHOLIC_BEVERAGE_DICHOTOMIZED	
CA1	FOOD_INTAKE_1	
CA1_DIC	FOOD_INTAKE_1_DICHOTOMIZED	
CA2	FOOD_INTAKE_2	
CA2_DIC	FOOD_INTAKE_2_DICHOTOMIZED	
CA3	FOOD_INTAKE_3	
CA3_DIC	FOOD_INTAKE_3_DICHOTOMIZED	
CA4	FOOD_INTAKE_4	
CA4_DIC	FOOD_INTAKE_4_DICHOTOMIZED	
CA5	FOOD_INTAKE_5	

CA5_DIC	FOOD_INTAKE_5_DICHOTOMIZED	
CA6	FOOD_INTAKE_6	
CA6_DIC	FOOD_INTAKE_6_DICHOTOMIZED	
PESO	WEIGHT	
EST	HEIGHT	
Estado_Nutricional	NUTRITIONAL_STATUS	
Estado_Nutricional_DIC	NUTRITIONAL_STATUS_DICHOTOMIZED	
CINT	WAIST_CIRCUMFERENCE	
CC_Categórico	WAIST_CIRCUMFERENCE_CATEGORISED	
Razão_Cintura_Estatura	WAIST_HEIGHT	
PAS1	SYSTOLIC_BLOOD_PRESSURE_1	
PAS2	SYSTOLIC_BLOOD_PRESSURE_2	
PAS3	SYSTOLIC_BLOOD_PRESSURE_3	
PAS_MÉDIA	SYSTOLIC_BLOOD_PRESSURE_AVERAGE	
PAD1	DIASTOLIC_BLOOD_PRESSURE_1	
PAD2	DIASTOLIC_BLOOD_PRESSURE_2	
PAD3	DIASTOLIC_BLOOD_PRESSURE_3	
PAD_MÉDIA	DIASTOLIC_BLOOD_PRESSURE_AVERAGE	
PA_CAT	BLOOD_PRESSURE_CATEGORISED	
PA_CAT_DIC	BLOOD_PRESSURE_DICHOTOMIZED	
ME1	HANDIGRIP_STRENGHT_LEFT_HAND_1	
ME2	HANDIGRIP_STRENGHT_LEFT_HAND_2	
ME3	HANDIGRIP_STRENGHT_LEFT_HAND_3	
MÉDIA_B	HANDIGRIP_STRENGHT_LEFT_HAND_AVERAGE	
MD1	HANDIGRIP_STRENGHT_RIGHT_HAND_1	
MD2	HANDIGRIP_STRENGHT_RIGHT_HAND_2	
MD3	HANDIGRIP_STRENGHT_RIGHT_HAND_3	
MÉDIA_C	HANDIGRIP_STRENGHT_RIGHT_HAND_AVERAGE	
TESTEFÍSICO	PYHSICAL_TEST	
COLETA1	BLOOD_COLLECTION_1	
COLETA2	BLOOD_COLLECTION_2	
COLETA3	BLOOD_COLLECTION_3	
COLETA4	BLOOD_COLLECTION_4	

COLETA5	BLOOD_COLLECTION_5	
COLETA6	BLOOD_COLLECTION_6	
COLETA7	BLOOD_COLLECTION_7	
PARQ	PHYSICAL_ACTIVITY_READINESS_QUESTIONNAIRE	
COL	TOTAL_CHOLESTEROL	
COL_CAT_BRA	TOTAL_CHOLESTEROL_CATEGORISED_BRAZIL	
COL_DIC_BRA	TOTAL_CHOLESTEROL_DICHOTOMIZED_BRAZIL	
COL_CAT	TOTAL_CHOLESTEROL_CATEGORISED	
HDL	HDL	
HDL_CAT_BRA	HDL_CATEGORISED_BRAZIL	
HDL_CAT	HDL_CATEGORISED	
LDL	LDL	
LDL_CAT_BRA	LDL_CATEGORISED_BRAZIL	
LDL_DIC_BRA	LDL_DICHOTOMIZED_BRAZIL	
LDL_CAT	LDL_CATEGORISED	
VLDL	VLDL	
TRI	TRIGLYCERIDES	
TRI_CAT_BRA	TRIGLYCERIDES_CATEGORISED_BRAZIL	
TRI_DIC_BRA	TRIGLYCERIDES_DICHOTOMIZED_BRAZIL	
TRI_CAT	TRIGLYCERIDES_CATEGORISED	
PCR	C_REACTIVE_PROTEIN	
PCR_NUM	C_REACTIVE_PROTEIN_VALUES	
PCR_CAT	C_REACTIVE_PROTEIN_CATEGORISED	
PCR_DIC	C_REACTIVE_PROTEIN__DICHOTOMIZED	
GLI	FASTING_GLUCOSE	
GLI_CAT	FASTING_GLUCOSE_CATEGORISED	
TOTALDEALUNOS	STUDENTS_TOTAL	
TV_Geral	WATCHING_TV_TOTAL	
VG_Celular_Geral	USING_TABLET_CELLPHONE_TOTAL	
Tela_TOTAL	TOTAL_SCREEN_TIME	
Tela_DIC_Q25	SCREEN_TIME_DICHOTOMIZED_25_QUARTILE	
Tela_DIC_Q75	SCREEN_TIME_DICHOTOMIZED_75_QUARTILE	
